# Socioeconomic support, quality of life, and prognosis of frailty among the older adults

**DOI:** 10.1002/hcs2.88

**Published:** 2024-03-25

**Authors:** Huai‐Yu Wang, Yuming Huang, Meng‐Ru Zhou, Hao‐Yue Jiang, Yu‐Han Zong, Xi‐Huan Zhu, Xiaojing Sun

**Affiliations:** ^1^ National Institute of Traditional Chinese Medicine Constitution and Preventive Treatment of Diseases Beijing University of Chinese Medicine Beijing China; ^2^ College of Chinese Medicine Beijing University of Chinese Medicine Beijing China; ^3^ Nephrology Department First Affiliated Hospital of Youjiang Medical College for Nationalities Baise China; ^4^ Institute of Basic Theory for Chinese Medicine China Academy of Chinese Medical Sciences Beijing China; ^5^ The Second School of Clinical Medicine Guangzhou University of Chinese Medicine Guangzhou China; ^6^ Qi‐huang Chinese Medicine School Beijing University of Chinese Medicine Beijing China; ^7^ Renal Division, Department of Medicine, Peking University First Hospital Peking University Institute of Nephrology Beijing China

**Keywords:** community support, delayed retirement, financial dependence, life satisfaction, progression of frailty

## Abstract

**Background:**

Although socioeconomic support is recommended for frailty management, its association with the prognosis of frailty is unclear.

**Methods:**

Using data from participants aged ≥65 years in the Chinese Longitudinal Healthy Longevity Survey (2008–2018), the associations between socioeconomic support (source of income, medical insurance, community support, living status), onset of prefrailty/frailty, and worsening of prefrailty, were analyzed using multinominal logistic regression models. The associations between self‐reported low quality of life (QoL) and reversion of prefrailty/frailty were analyzed using multivariate logistic regression models. Associations with mortality risk were analyzed using Cox proportional hazard regression models.

**Results:**

A total of 13,859 participants (mean age: 85.8 ± 11.1 years) containing 2056 centenarians were included. Financial dependence was a risk factor for low QoL among prefrail/frail individuals, but not among robust individuals. Having commercial or other insurance, and receiving social support from the community were protective factors for low QoL among prefrail/frail individuals and for the worsening of prefrailty. Continuing to work was a risk factor for low QoL, but a protective factor for worsening of prefrailty. A negative association between continuing to work and mortality existed in prefrail individuals aged <85 years and ≥85 years. Living alone was a risk factor for low QoL, but was not significantly associated with frailty prognosis.

**Conclusions:**

Prefrail and frail individuals were vulnerable to changes in socioeconomic support and more sensitive to it compared with robust individuals. Preferential policies regarding financial support, social support, and medical insurance should be developed for individuals with frailty.

## Graphical abstract

Based on the largest cohort of the longevous Chinese, the present study comprehensively evaluated the impacts of main contributors of socioeconomic support on the quality of life and prognosis of frailty. The prefrail and frail population were vulnerable to socioeconomic support and more sensitive to it than the robust population. Continuing to work at the advanced age was the risk factor of low quality of life but was the protective factor of worsening of prefrailty and survival. In sum, the present study provides evidence about socioeconomic support and prognosis of frailty for the clinical guidelines and policy makers. Frailty‐preferential policies on financial and social support and medical insurance should be developed.

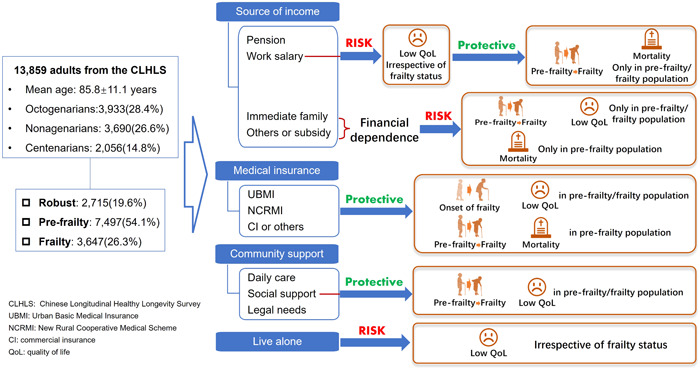

**Abbreviations**
95% CI: 95% confidence interval
CLHLS: Chinese Longitudinal Healthy Longevity Survey
HR: hazard ratio
ICFSR: International Conference of Frailty and Sarcopenia Research
IQR: interquartile range
NRCMS: New Rural Cooperative Medical Scheme
OR: odds ratio
QoL: quality of life
RRR: relative risk ratio
UBMI: Urban Basic Medical Insurance

## 1 INTRODUCTION

Frailty is recognized as having a major impact on healthy longevity worldwide. The prevalence of frailty continues to increase in parallel with the expansion of the aging population [1, 2, 3]. Previous studies reported that the amount of healthcare resources required by frail individuals is significantly greater than that required by robust individuals [2, 3, 4, 5, 6]. The reciprocal causation between frailty and various health conditions, such as multimorbidity and cognitive decline, aggravates the complexity of frailty management [3]. Efficient collaboration between families, communities, and wider society, and the optimized allocation of socioeconomic resources, are needed to support the management of frailty.

In recent years, increasing attention has been paid to socioeconomic support for frailty management [7, 8, 9, 10]. Although the International Conference of Frailty and Sarcopenia Research (ICFSR) strongly recommended that all individuals with frailty should be offered social support, they also reported that the impacts of socioeconomic support on the outcomes of frail elderly individuals, such as life satisfaction and prognosis of frailty, were unclear [11]. The guidelines of integrated care for older people established by the World Health Organization strongly recommended the establishment of a community and homecare‐dominated, public and private nursing home‐supplemented system for improving the care of older adults [12, 13]. The community and homecare‐dominated strategy clearly illustrated the importance of community support and living status (alone or not) for daily care of the older population. The importance of evaluating the social and medical care needs of older adults with or without frailty has also been highlighted [12, 13]. Overall, it is important to investigate the association between socioeconomic support, quality of life (QoL) and the prognosis of frailty. Such research could provide useful evidence for developing clinical guidelines of frailty management and the optimized allocation of socioeconomic resources to cope with the challenges faced by aging societies.

Thus, on the basis of a nationally representative cohort study of the older Chinese population with a 10‐year follow‐up period, using observational data of socioeconomic support and health, the present study explored the association between the main elements of socioeconomic support and self‐reported QoL, the transition of frailty, and mortality among individuals with and without frailty.

## 2 METHODS

### 2.1 Population

The Chinese Longitudinal Healthy Longevity Survey (CLHLS) is a nationally representative cohort study designed to investigate the determinants of healthy longevity in China [14, 15]. The CLHLS adopted a targeted random‐sample design, and recruited centenarians, nonagenarians, octogenarians, and younger residents living in the same region. Information including demographics, socioeconomic status, health status, and QoL was collected using structured questionnaires. Details of the CLHLS have been reported elsewhere [3, 16].

**Figure 1:**
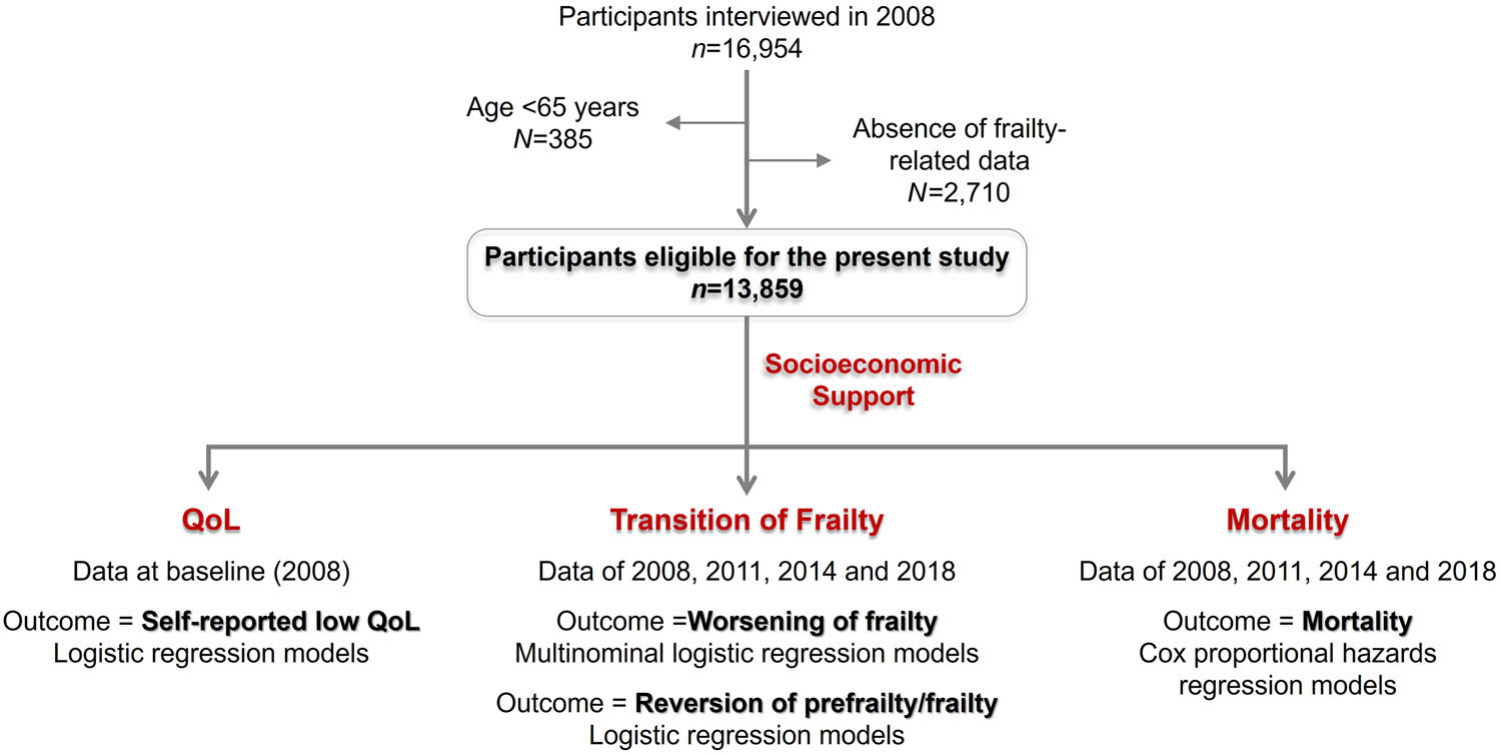
Flow chart of the present study.

The present study was conducted on the basis of the 2008 cohort, including data from interviews conducted in 2008, 2011, 2014 and 2018. A total of 16,954 participants were recruited at baseline (2008). Participants aged <65 years (*n* = 385) and those for whom frailty‐related data were absent in the 2008 interview (*n* = 2710) were excluded from the present analyses. Individuals excluded because of the absence of frailty‐related data were significantly older, with worse socioeconomic support and higher rates of low QoL (Table S1). Ultimately, 13,859 participants were eligible for inclusion in the present study (Figure 1). Information regarding the capacity for communication among centenarians was collected using a standard post‐interview questionnaire completed by the interviewer. These data are shown in Table S2.

The CLHLS was approved by the Research Ethics Committee of Peking University (IRB00001052‐13074). All participants provided written informed consent.

### 2.2 Variables

Levels of education were categorized into illiteracy, primary school, and middle school or above. Residence was recorded as urban or rural. Multimorbidity was defined as the self‐reported concurrent presence of three or more chronic conditions including hypertension, diabetes, heart disease, cerebrovascular disease, chronic pulmonary disease, eye disease, cancer, Parkinson's disease, dementia, mental illness, arthritis, gastrointestinal ulcer, hepatitis, and other conditions [3, 5, 17].

### 2.3 Socioeconomic support

Source of income was defined as the participants’ main source of economic resources, and was recorded as pension, continuing to work, dependence on immediate family members (spouse, child, or grandchild), and dependence on others or subsidies (relatives, friends, subsidies from government). Medical insurance was recorded as Urban Basic Medical Insurance (UBMI; including Urban Employee Basic Medical Insurance and Urban Resident Basic Medical Insurance), the New Rural Cooperative Medical Scheme (NRCMS), commercial insurance, and other insurance [18]. Community support was recorded as daily care (living care, shopping, home delivery), social support (spending time together, entertainment, social activities), legal needs (legal aid, mediation of disputes), daily care, social support, and all of the above. Living alone was recorded as a binary variable (Yes/No) by asking the question “Who do you live with? Family member, living alone, or living in a nursing home?”.

### 2.4 Identification of frailty

Frailty was defined using the modified Fried criteria [3, 5, 19]. Domains of frailty were defined as exhaustion (answered “always,” “often,” or “sometimes,” to “I felt old and useless” or “I felt everything I did was an effort”), weight loss (body mass index <18.5 kg/m^2^), weakness (inability to lift a bag weighing 5 kg), low mobility (inability to walk for 1 km) and inactivity (performing the following activities ≤1 time/week: housework, outside activity, gardening, keeping a pet, raising domestic animals, playing cards or mah‐jong, and social activity) [5, 20, 21, 22]. Participants meeting ≥3 domains were defined as frail, those meeting 1–2 domains were defined as pre‐frail, and those meeting none of the domains were defined as robust.

### 2.5 Outcomes

Self‐reported QoL at baseline (2008) was recorded by asking “what do you think about your life?” [23, 24]. Participants who answered “fair”, “bad,” or “very bad” were classified as having low QoL.

Participants with data regarding frailty in 2011, 2014, and 2018 were included in the analyses of the transition of frailty. The transition of frailty was defined as persistent status or altered status during follow‐up in reference to frailty status at the first interview, as follows: (1) persistent robust status from baseline to the end‐point; (2) onset of prefrailty or frailty during the follow‐up period; (3) progression from prefrailty to frailty during the follow‐up period; (4) persistent prefrail/frail status from the baseline to the end‐point; (5) reversion from prefrail to robust or reversion from frail to prefrail/robust.

All‐cause mortality was recorded. The follow‐up duration was 55 months (interquartile range [IQR]: 25–95 months).

### 2.6 Statistics

Characteristics and outcomes of the study population were presented by the status of frailty at baseline and compared using one‐way analysis of variance and chi square tests. The proportions of participants with different types of medical insurance under different sources of income were described by frailty status.

The association between socioeconomic support and the odds of self‐reported low QoL for each frailty status were analyzed with multivariate logistic regression models using baseline data for socioeconomic support and QoL. Covariates including age, sex, education, residence (urban/rural), and multimorbidity were adjusted. Odds ratios (ORs) with 95% confidence intervals (CIs) were calculated.

Among the population with persistent robust status (as a reference), showing the onset of pre‐frailty/frailty and worsening of pre‐frailty to frailty, the association between socioeconomic support and the risk of onset of prefrailty/frailty, as well as the risk of worsening of prefrailty were analyzed using multinominal logistic regression models, using individuals with persistent robust status as a reference. The adjusted covariates were the same as those mentioned above. The results were presented as relative risk ratios (RRRs) with 95% CIs. Among the population with persistent pre‐frailty/persistent frailty status (as a reference) and those with reversion from pre‐frail to robust status, or frail to pre‐frail/robust status, the association between socioeconomic support and the probability of reversion of prefrail/frail status were analyzed using multivariate logistic regression models. The adjusted covariates were the same as those mentioned above. ORs with 95% CIs were calculated.

Cox proportional hazards regression models were adopted to evaluate the association between socioeconomic support and the risk of mortality stratified by frailty status. Baseline data of socioeconomic support were used in the analyses. Results were presented as hazard ratios (HRs) with 95% CI. Considering the influence of age on the association between socioeconomic status and survival, age‐stratified analyses (<85 years and ≥85 years) were performed for each frailty status.

All analyses were two‐tailed and the level of statistical significance was set at *p* < 0.05. Stata version 16.0 (Stata Corp LP, College Station, TX, USA) was used for all statistical analyses.

## 3 RESULTS

### 3.1 Population characteristics

A flow chart of the present study design is shown in Figure 1. A total of 13,859 adults (85.8 ± 11.1 years) were recruited, including 2056 (14.8%) centenarians, 3690 (26.6%) nonagenarians, and 3933 (28.4%) octogenarians. Among them, 2715 (19.6%) individuals were categorized as robust, 7497 (54.1%) were prefrail, and 3647 (26.3%) were frail. Rates of financial dependence increased with the severity of frailty. A total of 51.7% of the robust population, 72.4% of the prefrail population and 88.0% of the frail population lived on financial support from their immediate family, or others, or subsidy. A total of 16.0% of the robust population and 10.8% of the prefrail population still worked for living (Table 1). The demographic characteristics of centenarians are shown in Table S3.

**Table 1:** Demographic characteristics and prognosis of frailty, by frailty status at baseline.

Characteristics	Overall	Robust	Prefrailty	Frailty	*P* value
In total (%)	13,859 (100.0)	2715 (19.6)	7497 (54.1)	3647 (26.3)	
Age (mean ± SD)	85.8 ± 11.1	79.2 ± 10.0	84.2 ± 10.5	93.8 ± 8.2	
Sex, *n* (%)					<0.001
Male	6252 (45.1)	1538 (56.7)	3593 (47.9)	1121 (30.7)	
Female	7607 (54.9)	1177 (43.4)	3904 (52.1)	2526 (69.3)	
Education, *n* (%)					<0.001
Illiteracy	8279 (59.7)	1110 (40.9)	4421 (59.0)	2748 (75.4)	
Primary school	4082 (29.5)	1081 (39.8)	2311 (30.8)	690 (18.9)	
Middle school or above	1498 (10.8)	524 (19.3)	765 (10.2)	209 (5.7)	
Residence, *n* (%)					<0.001
Urban	5648 (40.8)	1295 (47.7)	2814 (37.5)	1539 (42.2)	
Rural	8211 (59.3)	1420 (52.3)	4683 (62.5)	2108 (57.8)	
Multimorbidity, *n* (%)					<0.001
1–2 diseases	5984 (46.4)	1165 (45.1)	3181 (45.6)	1638 (48.9)	
≥3 diseases	1036 (8.0)	178 (6.9)	522 (7.5)	336 (10.0)	
Source of income, *n* (%)					<0.001
Pension	2551 (18.4)	877 (32.3)	1260 (16.8)	414 (11.4)	
Continuing to work	1271 (9.2)	434 (16.0)	813 (10.8)	24 (0.7)	
Immediate family	8404 (60.6)	1202 (44.3)	4625 (61.7)	2577 (70.7)	
Others or subsidy	1633 (11.8)	202 (7.4)	799 (10.7)	632 (17.3)	
Medical insurance, *n* (%)					<0.001
UBMI	3071 (22.2)	940 (34.6)	1513 (20.2)	618 (17.0)	
NRCMS	6584 (47.5)	1087 (40.0)	3827 (51.1)	1670 (45.8)	
Commercial or others	535 (3.9)	123 (4.5)	245 (3.3)	167 (4.6)	
None	3669 (26.5)	565 (20.8)	1912 (25.5)	1192 (32.7)	
Community Support, *n* (%)					<0.001
Daily care	532 (3.9)	95 (3.5)	272 (3.6)	165 (4.5)	
Socializing	661 (4.8)	176 (6.5)	326 (4.4)	159 (4.4)	
Legal needs	1868 (13.5)	357 (13.2)	1082 (14.5)	429 (11.8)	
Daily care & socializing	339 (2.5)	68 (2.5)	175 (2.3)	96 (2.6)	
All above	525 (3.8)	120 (4.4)	272 (3.6)	133 (3.7)	
Don't know or none	9895 (71.6)	1887 (69.8)	5349 (71.6)	2659 (73.0)	
Live alone, *n* (%)	2304 (16.6)	400 (14.7)	1461 (19.5)	443 (12.2)	<0.001
Low QoL, *n* (%)	5642 (40.8)	784 (28.9)	3256 (43.5)	1602 (44.0)	<0.001
Transition of frailty*, *n* (%)					–
Persistent robustness	–	364 (20.1)	–	–	
Onset of prefrailty or frailty	–	1121 (61.9)	–	–	
Progress from prefrailty to frailty	–	–	722 (17.9)	–	
Reverse to robustness or prefrailty	–	–	897 (22.2)	387 (46.2)	
Persistent prefrailty/frailty	–	–	1754 (43.5)	417 (49.8)	
Mortality, *n* (%)	7632 (65.7)	1042 (45.5)	3957 (62.0)	2633 (89.3)	<0.001
Follow‐up duration (month, median[quartile])	55 (25, 95)	71 (42, 118)	65 (30, 106)	28 (14, 54)	<0.001

Abbreviations: NRCMS, New Rural Cooperative Medical Scheme; QoL, quality of life; UBMI, Urban Basic Medical Insurance.

*Note*: *Rates of transition of frailty were calculated among the population having frailty data during the interviews in 2011, 2014, and 2018. The numbers of individuals with frailty data during follow‐up were as follows: robust 1812, prefrail 4035, frail 837.

Irrespective of frailty status, the highest rates of having UBMI (52.3% to 69.9%) were observed among participants that drew a pension. The highest rates of having NCRMS (63.1% to 82.2%) were observed among individuals who were financially dependent on their immediate family members. The highest rates of having commercial insurance or subsidies in robust individuals were among those drawing a pension, whereas the highest proportions in prefrail and frail individuals were among those who were financially dependent on their immediate family members (Table S4).

### 3.2 Socioeconomic support and self‐reported low QoL

**Figure 2:**
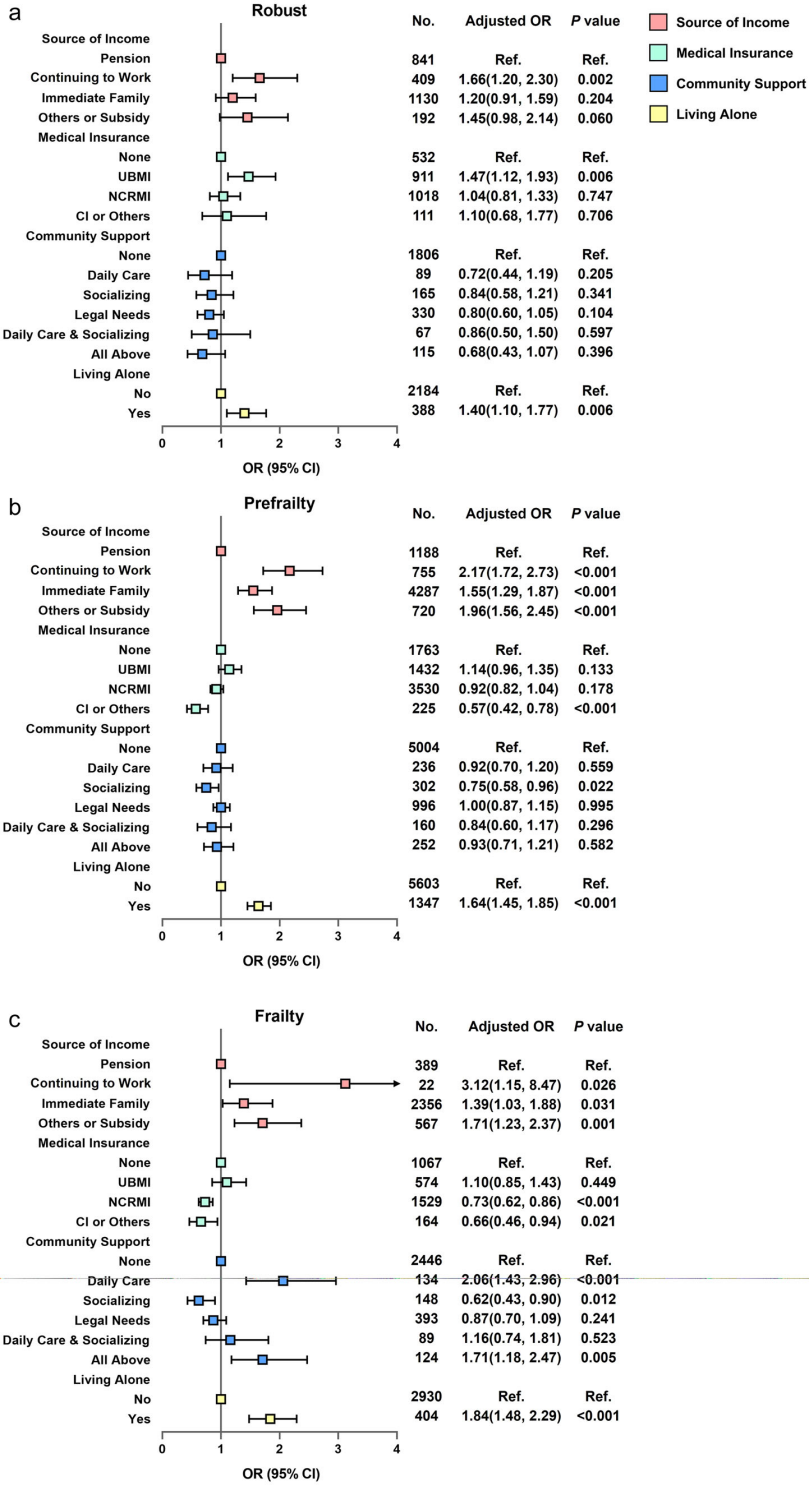
Association between socioeconomic support and the odds of low QoL at baseline, stratified by frailty status. Adjusted for age, sex, education, residence (urban/rural), and multimorbidity. (a) Robust population; (b) Prefrail population; (c) Frail population. CI, commercial insurance; NRCMS, New Rural Cooperative Medical Scheme; QoL, quality of life; UBMI, Urban Basic Medical Insurance.

A total of 5642 (40.8%) individuals reported low QoL at baseline (Table 1). Financial dependence (depending on immediate family members, or others, or subsidies) was significantly associated with increased odds of low QoL among the population with prefrailty or frailty, but not among the robust population. Continuing to work and living alone showed a significant association with increased odds of low QoL, irrespective of frailty status. Having commercial or other insurance and receiving social support from the community were associated with decreased odds of low QoL among prefrail and frail individuals, but not among robust individuals (Figure 2).

### 3.3 Socioeconomic support and transition of frailty

The results revealed that 1121 (61.9%) robust participants developed prefrailty or frailty, and 722 (17.9%) prefrail participants progressed to frailty during the follow‐up period (Table 1).

**Figure 3:**
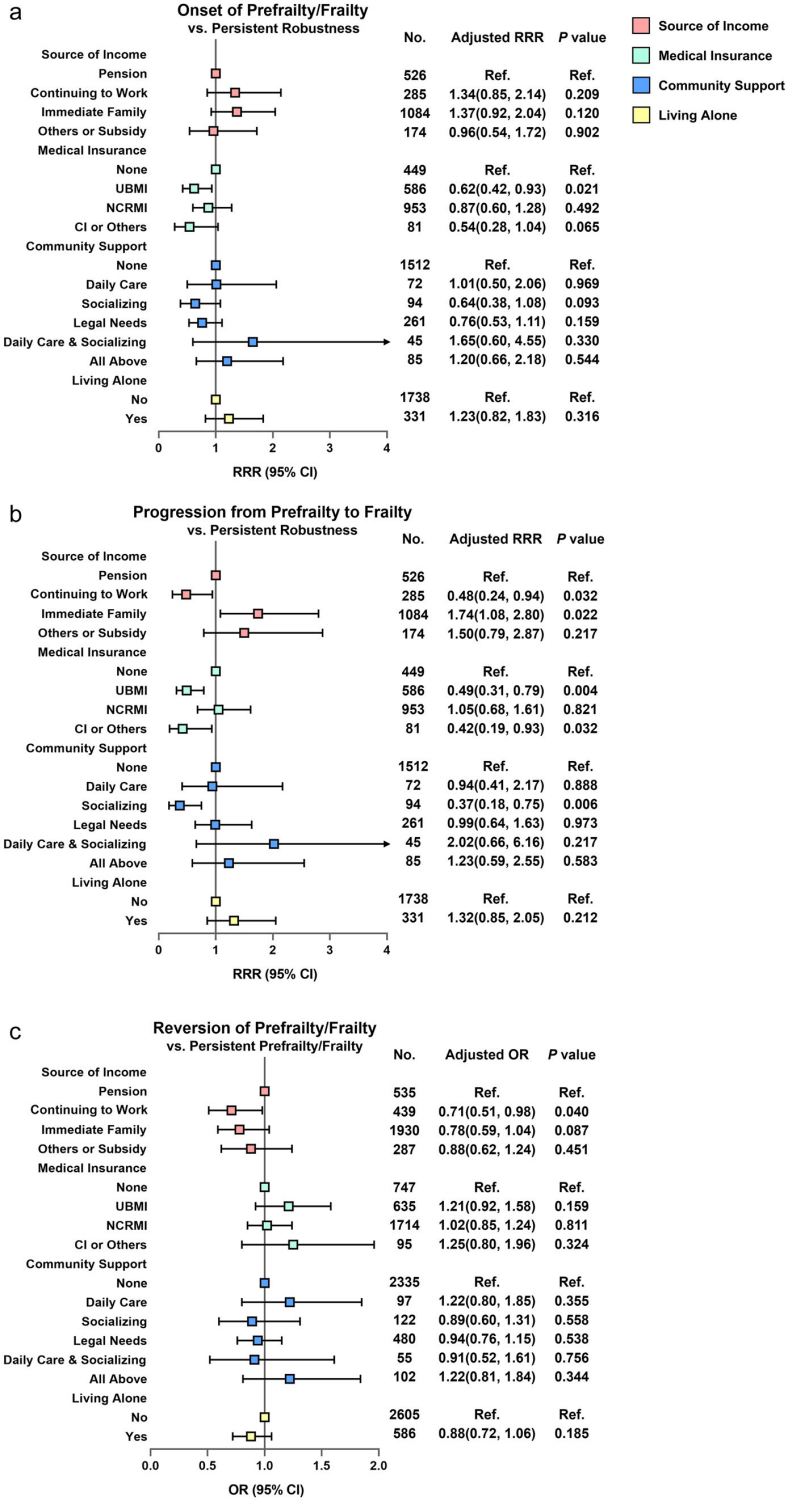
Association between socioeconomic support and transition of frailty. (a) Association between socioeconomic support and the risk of onset of prefrailty/frailty. Multinominal logistic regression models, onset of prefrailty/frailty in reference to individuals with persistent robust status. (b) Association between socioeconomic support and the risk of worsening of prefrailty. Multinominal logistic regression models, worsening of prefrailty in reference to individuals with persistent robust status. Age, sex, education, residence (urban/rural), and multimorbidity were adjusted as confounders. (c) Association between socioeconomic support and the probability of reversion of prefrailty/frailty, in reference to individuals with persistent pre‐frail/frail status. Logistic regression models, age, sex, education, residence (urban/rural) and multimorbidity were adjusted as confounders. CI, commercial insurance; NRCMS, New Rural Cooperative Medical Scheme; QoL, quality of life; RRR, relative risk ratio; UBMI, Urban Basic Medical Insurance.

Having UBMI was significantly associated with a reduced risk of the onset of prefrailty/frailty. Continuing to work, having UBMI, having commercial or other insurance and receiving socializing support from the community were significantly associated with a reduced risk of worsening prefrailty. Financial dependence on immediate family members was associated with an increased risk of worsening of prefrailty (Figure 3a–b).

The results revealed that 897 (22.2%) participants with prefrailty and 387 (46.2%) participants with frailty reverted to robust or prefrail status during the follow‐up period (Table 1). Continuing to work was significantly associated with a reduced probability of reversion of prefrailty/frailty. No significant relationships were observed among other socioeconomic support factors and the probability of reversion of prefrailty/frailty (Figure 3c).

### 3.4 Socioeconomic support and mortality

**Figure 4:**
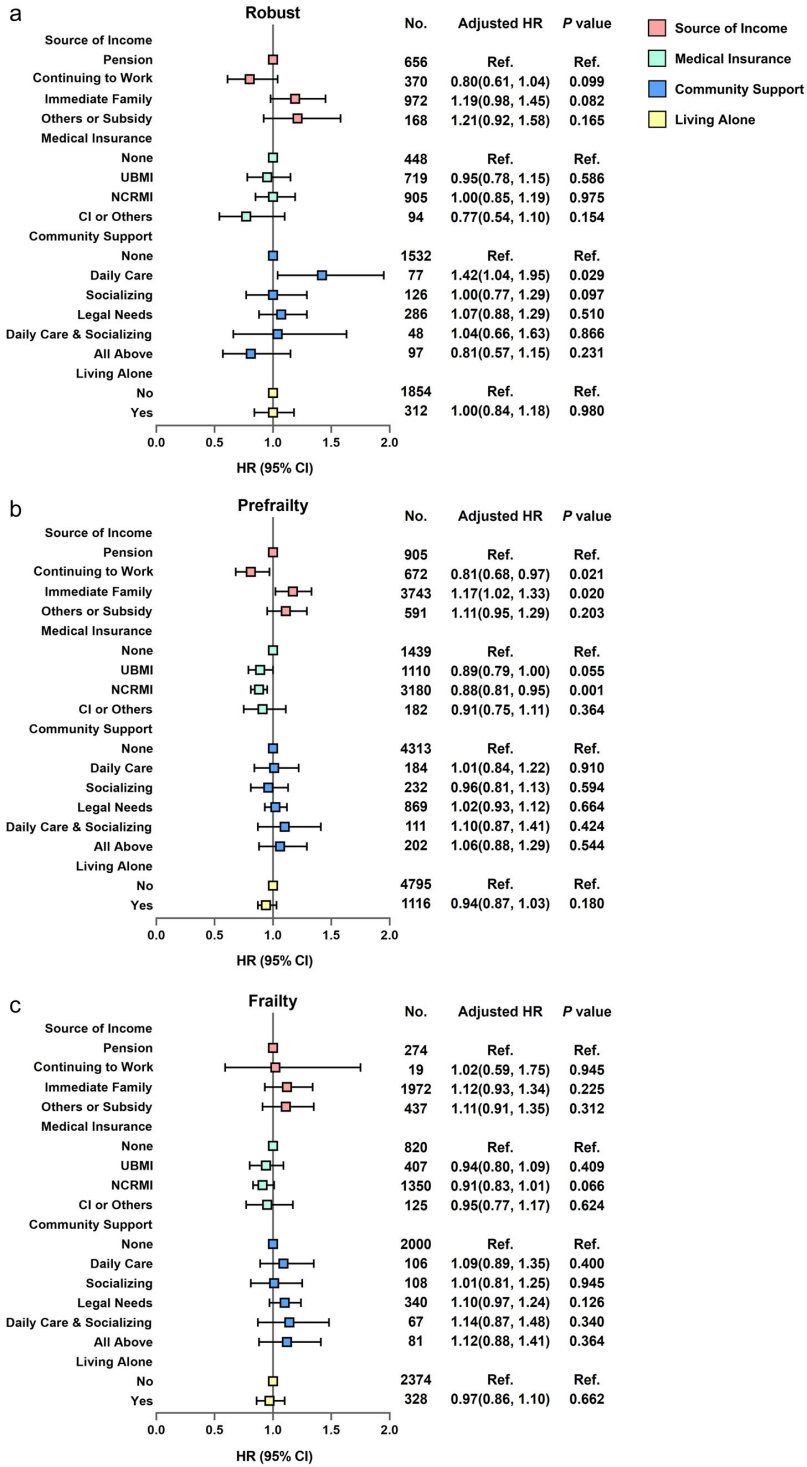
Association between socioeconomic support and the risk of mortality, stratified by frailty status. Adjusted for age, sex, education, residence (urban/rural) and multimorbidity. (a) Robust population; (b) Prefrail population; (c) Frail population. CI, commercial insurance; NRCMS, New Rural Cooperative Medical Scheme; QoL, quality of life; UBMI, Urban Basic Medical Insurance.

In total, 1042 (45.5%) robust participants, 3957 (62.0%) prefrail participants, and 2633 (89.3%) frail participants died during the follow‐up period (Table 1). Among the prefrail population, continuing to work and having NRCMS were significantly associated with the reduced risk of mortality, while financial dependence on immediate family members was associated with an increased risk of mortality. No similar results were found among the robust and frail populations (Figure 4).

A significant association between continuing to work and the reduced risk of mortality in the prefrail population existed among individuals aged <85 years and those aged ≥85 years. Similar results were observed among the robust population aged <85 years. The significant association between financial dependence and the increased risk of mortality were observed in the robust population aged ≥85 years and in the prefrail population aged ≥85 years. Living alone was a risk predictor of mortality among the robust population younger than 85 years, but was associated with a reduced risk of mortality among the prefrail population aged ≥85 years (Table S5).

## 4 DISCUSSION

In a large cohort of the older Chinese population, the present study provided evidence regarding the relationship between socioeconomic support and the prognosis of frailty. These findings may be valuable for policy makers and the development of clinical guidelines. The results revealed a negative association between having medical insurance and an increased risk of low QoL and progression of frailty, as well as a positive association between financial dependence and an increased risk of low QoL and progression of frailty, in prefrail and frail individuals. This pattern may be partially explained by the increased medical needs of these populations. Notably, continuing to work for living at an advanced age was a risk factor for low QoL, but was a protective factor for the worsening of prefrailty and survival. Living alone was a risk factor for low QoL but had a nonsignificant association with the prognosis of frailty. Social support from the community was a protective factor for QoL and the progression of frailty among the prefrail/frail population.

Financial dependence of the older adults is an important issue that significantly impacts self‐reported QoL and the prognosis of frailty. In the current study population (mean age: 85 years), only 32.3% of robust participants, 16.8% of prefrail participants, and 11.4% of frail participants lived on a pension without any financial dependence on other individuals. Generally, levels of pension are calculated on the basis of the levels of salary and types of occupation. The financial security associated with high‐income occupations translated into larger pensions and influenced the QoL of elderly individuals. Consistent with the present results, Dugravot et al. provided evidence from France reporting that the financial protection of health of individuals with higher‐income occupations at age 50 years was associated with a reduced risk of frailty in advanced age [7]. Irrespective of the differences in social and financial environments between countries, financial security has a substantial impact on healthy aging. More than half of the current study population were financially dependent on their immediate family members. The significant correlation between financial dependence and low QoL suggests that financial dependence has adverse impacts on life satisfaction. In China, efforts and shortcomings in equalizing financial protection could provide a reference for other developing countries. The Chinese government issued over 30 national policies during the past decade to modify the pension and subsidy schemes [12]. As of October 2021, more than half of China's provincial‐level regions had raised the payment standard of the old‐age pension covering over 72 million elderly individuals in urban and rural areas [25]. However, implementing a unified level of subsidy or an identical subsidy calculation scheme for all older adults is suboptimal, particularly for cash‐strapped local governments. For example, Guangxi is the province with the densest population of oldest‐old individuals in China, but is ranked 19th of 31 provinces in China for gross domestic product. Considering the different associations between financial support and outcomes in populations with or without frailty, individualized financial support in accordance with frailty status may be more feasible and cost‐efficient to implement in developing regions like Guangxi Province.

The opposite trends of associations between continuing to work, self‐reported QoL, and the prognosis of frailty in the older adults should be noted. The positive association between continuing to work and low QoL was consistent with a previous study reporting that retirement can significantly improve the happiness of elderly individuals [26]. Continuing to work at an advanced age is likely to be stressful, potentially resulting in low QoL. However, it should be emphasized that continuing to work was significantly associated with better survival. Frailty is characterized as a deficit in physical function, manifested as inactivity, low mobility, and weakness [1, 4, 11]. The ICFSR guidelines recommended multicomponent physical activity programs as first‐line therapy for frailty [11]. Although there are substantial differences between performing work and performing physical training, engaging in work typically increases physical and cognitive activity and social interaction. Mäcken et al. reported faster memory decline after retirement using data from the Survey of Health, Aging, and Retirement in Europe [27]. Dave et al. reported a 5%–16% increase in difficulties associated with mobility and daily activities and a 5%–6% increase in illness conditions over an average postretirement period of 6 years [28]. Bozio et al. reported that no significant increase of mortality was found with delayed retirement [29]. In accord with the adage “use it or lose it,” continuing to work may be helpful for maintaining physical and cognitive functions against the functional deterioration that occurs with aging. A similar phenomenon may partially explain the present results regarding living alone. Living alone was found to be more likely to expose individuals to social isolation compared with living with others, potentially causing an increased risk of unsatisfactory QoL. This is consistent with results of a previous systematic review reporting living alone as a risk factor for depression, particularly in studies with a cross‐sectional design [30]. Meanwhile, a previous meta‐analysis reported that living alone was positively associated with an increased risk of frailty in a cross‐sectional study, but not in a longitudinal study [31]. This finding is consistent with the present results. Living alone forces older adults to care for themselves in daily life, which often involves an increase in physical and cognitive activity. This could potentially explain the lack of a significant association between living alone and the prognosis of frailty. The improvement of delayed retirement policy is an important issue faced by many countriesg [32, 33]. The present results suggest that prefrailty should not be an exclusive criterion for delayed retirement. However, given concerns about accumulated physical strain, which may explain why continuing to work was a risk factor for the persistence of prefrailty/frailty in the present study, occupations targeted for delayed retirement should be carefully selected.

Health insurance is correlated to financial support and health. In the present study, the results revealed a negative association between commercial or other insurance and an increased risk of low QoL among prefrail/frail individuals, but not among robust individuals. In China, social basic medical insurance is the predominant health insurance scheme with the coverage rate of 100% [18]. Generally, individuals pay for commercial insurance to supplement social insurance, and those with catastrophic medical expenditure and/or those who are living at or below the poverty line are subsidized. Among those with commercial insurance and those receiving subsidies in the present study, 25.2% of robust individuals, 46.5% of prefrail individuals, and 59.9% of frail individuals were financially dependent on their immediate family members. High rates of commercial insurance or subsidy were associated with increased demands of reimbursement and subsidization with the progression of frailty, indicating that the current social insurance system is unsatisfactory for frail older individuals. It should be noted that the present results showed an association between Chinese social health insurance and healthy aging. Thus, these results might not be reproduced in other societies with different health insurance schemes. Although health insurance schemes differ between countries, the increased medical and reimbursement demands of frail populations are substantial. Preferential policies for reimbursement in accord with frailty status should be developed.

Support from the community is an important factor in frailty management [10]. Empirical evidence indicates that care in daily living is the most essential type of support for elderly individuals. However, the present results indicated that social support, rather than daily care, was associated with a reduced risk of low QoL and worsening of prefrailty. Because an observational study design was used, the present study was not able to confirm the effectiveness of community support for improving QoL and the transition of frailty. However, previous randomized controlled trials conducted in Austria reported that home‐based volunteer‐administered social support alone (getting out, having a cha‐t, or sharing interests) could effectively improve QoL and frailty status [10, 34]. Taken together, these findings suggest that enhancement of social contact in the community may be beneficial for the management of frailty. For low‐income regions, social support could be developed preferentially if socioeconomic resources are insufficient to establish a comprehensive community support system.

The present study involved several limitations. First, because of the nature of the observational study design, the present study was exploratory, and causal conclusions could not be drawn. Second, the status of frailty during the follow‐up period was assessed in interviews, and the exact dates of frailty transitions were unavailable. Third, although the study had a large sample size of oldest‐old individuals, the frequency of data for some items in specific categories was low. Fourth, the CLHLS integrated data from the Urban Employee Basic Medical Insurance and Urban Resident Basic Medical Insurance as UBMI, although the target populations and reimbursement policies of these schemes are different. Fifth, because of the relatively high rate of low QoL (40.8%), the association between socioeconomic support and odds of low QoL may have been overestimated. Finally, the possibility of residual confounding cannot be ruled out.

## 5 CONCLUSION

Pre‐frail and frail elderly individuals were found to be more vulnerable to changes of socioeconomic support compared with robust individuals, possibly because of their greater medical demands. Preferential policies for financial support, medical insurance, and community support are needed to improve self‐reported QoL and the prognosis of frailty.

## AUTHOR CONTRIBUTIONS

Huai‐Yu Wang participated in research design, funding acquisition, data acquisition, analyses and interpretation, manuscript drafting and revision. Yuming Huang participated in data interpretation and manuscript revision. Meng‐Ru Zhou, Hao‐Yue Jiang, Yu‐Han Zong and Xi‐Huan Zhu participated in data analyses and manuscript revision. Xiaojing Sun participated in data interpretation, funding acquisition, and manuscript revision.

## CONFLICT OF INTEREST STATEMENT

The authors declare no conflicts of interest.

## ETHICS STATEMENT

The present study was conducted based on the CLHLS. The CLHLS was approved by the Research Ethics Committee of Peking University (IRB00001052‐13074).

## INFORMED CONSENT

All participants provided written informed consent.

## Supporting information

Supporting information.

## Data Availability

The datasets generated during and/or analyzed during the current study is from a publicly available source, which is the CLHLS repository and could be accessed by: https://opendata.pku.edu.cn/dataverse/CHADS.
