# Supplementary material for: Socioeconomic support, quality of life, and prognosis of frailty among the older adults
Source: Health Care Sci. 2024 Mar 25;3(2):101–13. doi: 10.1002/hcs2.88 (PMC11080858; doi:10.1002/hcs2.88)
Supplement: Supplementary file 1 — Supporting information. [file HCS2-3-101-s001.docx]

**Supplementary Material**

**Table S.1. Characteristics comparison between individuals included in the present study and excluded due to the absence of frailty-related data.**

| **Characteristics** | **Excluded** | **Included** | ***P* value** |
| --- | --- | --- | --- |
| In total (%) | 2710 | 13859 |  |
| Age (mean±SD) | 96.2±8.0 | 85.8±11.1 | <0.001 |
| Sex (n, %) |  |  | <0.001 |
| Male | 740(27.3) | 6252(45.1) |  |
| Female | 1970(72.7) | 7607(54.9) |  |
| Education (n, %) |  |  | <0.001 |
| Illiteracy | 2187(80.7) | 8279(59.7) |  |
| Primary school | 406(15.0) | 4082(29.5) |  |
| Middle school or above | 116(4.3) | 1498(10.8) |  |
| Residence (n, %) |  |  | <0.001 |
| Urban | 936(34.5) | 5648(40.8) |  |
| Rural | 1774(65.5) | 8211(59.3) |  |
| Source of income (n, %) |  |  | <0.001 |
| Pension | 245(9.0) | 2551(18.4) |  |
| Continuing to Work | 30(1.1) | 1271(9.2) |  |
| Immediate Family | 1878(69.3) | 8404(60.6) |  |
| Others or Subsidy | 557(20.6) | 1633(11.8) |  |
| Medical insurance (n, %) |  |  | <0.001 |
| UBMI | 378(14.0) | 3071(22.2) |  |
| NCRMI | 1480(54.6) | 6584(47.5) |  |
| CI or Others | 132(4.9) | 535(3.9) |  |
| None | 719(26.5) | 3669(26.5) |  |
| Community Support (n, %) |  |  | <0.001 |
| Daily Care | 109(4.0) | 532(3.9) |  |
| Socializing | 117(4.3) | 661(4.8) |  |
| Legal Needs | 326(12.0) | 1868(13.5) |  |
| Daily Care & Socializing | 80(3.0) | 339(2.5) |  |
| All Above | 51(1.9) | 525(3.8) |  |
| None | 2024(74.8) | 9895(71.6) |  |
| Live alone (n, %) | 242(8.9) | 2304(16.6) | <0.001 |
| Low QoL (n, %) | 2297(84.8) | 5642(40.8) | <0.001 |

Abbreviations: QoL: quality of life; UBMI: Urban Basic Medical Insurance; NRCMS: New Rural Cooperative Medical Scheme; CI: commercial insurance; QoL: quality of life.

**Table S.2. Capacity of communication, among the centenarians.**

| Items | No. | Frequency (%) |
| --- | --- | --- |
| Was the interviewee able to hear? |  |  |
| Yes, without hearing aid | 928 | 45.14 |
| Yes, but needs hearing aid | 308 | 14.98 |
| Partly, despite hearing aid | 633 | 30.79 |
| No | 187 | 9.10 |
| Did the interviewee able to participate in physical check? |  |  |
| Yes | 1344 | 65.37 |
| No | 149 | 7.25 |
| Partially able to | 563 | 27.38 |
| The health of interviewee rated by interviewer |  |  |
| Surprisingly healthy | 329 | 16.00 |
| Relatively healthy | 1327 | 64.54 |
| Moderately ill | 370 | 18.00 |
| Very ill | 30 | 1.46 |
| Has interviewer checked for failure to ask a question? |  |  |
| Yes | 1990 | 96.79 |
| No | 66 | 3.21 |
| Did anyone help the interviewee to answer question |  |  |
| Yes | 1643 | 79.91 |
| No | 413 | 20.09 |
| Who helped the interviewee to answer questions? |  |  |
| Spouse | 31 | 1.89 |
| Child or spouse of child | 1176 | 71.58 |
| Grandchild or spouse of grandchild | 297 | 18.08 |
| Great grandchild or spouse of great grandchild | 35 | 2.13 |
| Sibling | 1 | 0.06 |
| Parent or parent-in-law | 50 | 3.04 |
| Caregiver or institutional staff | 53 | 3.23 |
| Others | 413 | 1.89 |

**Table S. 3. Demographic characteristics of centenarians.**

| **Characteristics** | **Robust** | **Prefrailty** | **Frailty** | ***P* value** |
| --- | --- | --- | --- | --- |
| In total (%) | 103(5.0) | 741(36.0) | 1212(59.0) |  |
| Age (mean± SD) | 102.0±2.6 | 102.0±2.1 | 102.4±2.3 | 0.001 |
| Sex (n, %) |  |  |  | <0.001 |
| Male | 44(42.7) | 204(27.5) | 222(18.3) |  |
| Female | 59(57.3) | 537(72.5) | 990(81.7) |  |
| Education (n, %) |  |  |  | 0.056 |
| Illiteracy | 80(77.7) | 597(80.6) | 1031(85.1) |  |
| Primary school | 17(16.5) | 113(15.3) | 142(11.7) |  |
| Middle school or above | 6(5.8) | 31(4.2) | 39(3.2) |  |
| Residence (n, %) |  |  |  | 0.006 |
| Urban | 38(36.9) | 265(35.8) | 520(42.9) |  |
| Rural | 65(63.1) | 476(64.2) | 692(57.1) |  |
| Multimorbidity (n, %) |  |  |  | <0.001 |
| 1~2 diseases | 38(36.9) | 249(35.3) | 506(45.3) |  |
| ≥3 diseases | 6(5.8) | 31(4.4) | 74(6.6) |  |
| Source of income (n, %) |  |  |  | <0.001 |
| Pension | 21(20.4) | 51(6.9) | 74(6.1) |  |
| Continuing to Work | 2(1.9) | 4(0.5) | 1(0.1) |  |
| Immediate Family | 59(57.3) | 503(67.9) | 814(67.2) |  |
| Others or Subsidy | 21(20.4) | 183(24.7) | 323(26.7) |  |
| Medical insurance (n, %) |  |  |  | <0.001 |
| UBMI | 24(23.3) | 98(13.2) | 188(15.5) |  |
| NCRMI | 47(45.6) | 410(55.3) | 538(44.4) |  |
| CI or Others | 11(10.7) | 57(7.7) | 81(6.7) |  |
| None | 21(20.4) | 176(23.8) | 405(33.4) |  |
| Community Support (n, %) |  |  |  | 0.103 |
| Daily Care | 2(1.9) | 32(4.3) | 64(5.3) |  |
| Socializing | 14(13.6) | 50(6.8) | 82(6.8) |  |
| Legal Needs | 15(14.6) | 82(11.1) | 136(11.2) |  |
| Daily Care & Socializing | 6(5.8) | 24(3.2) | 41(3.4) |  |
| All Above | 4(3.9) | 22(3.0) | 52(4.3) |  |
| None | 62(60.2) | 531(71.7) | 837(69.1) |  |
| Live alone (n, %) | 16(15.5) | 98(13.2) | 84(6.9) | <0.001 |
| Low QoL (n, %) | 18(17.5) | 255(34.6) | 453(37.5) | <0.001 |
| Transition of Frailty * (n, %) |  |  |  | <0.001 |
| Persistent robustness | 8(19.5) | / | / |  |
| Onset of prefrailty or frailty | 31(75.6) | / | / |  |
| Progress from prefrailty to frailty | / | 59(41.0) | / |  |
| Reverse to robustness or prefrailty | / | 9(6.3) | 59(37.6) |  |
| Persistent prefrailty/frailty | / | 58(40.3) | 95(60.5) |  |
| Mortality (n, %) | 68(79.1) | 544(91.0) | 904(94.2) | <0.001 |
| Follow-up duration (month, median[quartile]) | 44.5(28, 67) | 27(14, 49) | 23(10, 38) | <0.001 |

* Rates of transition of frailty were calculated among the population having data of frailty during the interviews in 2011, 2014 and 2018. The numbers of individual having data of frailty during the follow-up were: robust 41, prefrailty 144, frailty 157.

Abbreviations: QoL: quality of life; UBMI: Urban Basic Medical Insurance; NRCMS: New Rural Cooperative Medical Scheme; CI: commercial insurance; QoL: quality of life.

**Table S. 4. Type of medical insurance in different sources of income, stratified by the status of frailty.**

| **Medical Insurance** | **Pension** | **Continuing to Work** | **Immediate Family** | **Others or Subsidy** |
| --- | --- | --- | --- | --- |
| ***Robust*** |  |  |  |  |
| None | 103(18.2) | 120(21.2) | 293(51.9) | 49(8.7) |
| UBMI | 657(69.9) | 49(5.2) | 192(20.4) | 42(4.5) |
| NRCMS | 59(5.4) | 251(23.1) | 686(63.1) | 91(8.4) |
| Commercial or Others | 58(47.2) | 14(11.4) | 31(25.2) | 20(16.3) |
| ***Prefrailty*** |  |  |  |  |
| None | 179(9.4) | 224(11.7) | 1317(68.9) | 192(10.0) |
| UBMI | 936(61.9) | 50(3.3) | 418(27.6) | 109(7.2） |
| NRCMS | 107(2.8) | 531(13.9) | 2776(72.5) | 413(10.8) |
| Commercial or Others | 38(15.5) | 8(3.3) | 114(46.5) | 85(34.7) |
| ***Frailty*** |  |  |  |  |
| None | 57(4.8) | 7(0.6) | 915(76.8) | 213(17.9) |
| UBMI | 323(52.3) | 1(0.2) | 189(30.6) | 105(17.0) |
| NRCMS | 20(1.2) | 16(1.0) | 1373(82.2) | 261(15.6) |
| Commercial or Others | 14(8.4) | 0(0.0) | 100(59.9) | 53(31.7) |

Abbreviations: UBMI: Urban Basic Medical Insurance; NRCMS: New Rural Cooperative Medical Scheme.

**Table S. 5. Association between socioeconomic support and the risk of mortality, frailty- and age- stratified.** Adjusted for sex, residence (urban/rural), education and multimorbidity.

| Variables | Robust | | | | Prefrailty | | | | Frailty | | | |
| --- | --- | --- | --- | --- | --- | --- | --- | --- | --- | --- | --- | --- |
|  | age<85 years | | age≥85 years | | age<85 years | | age≥85 years | | age<85 years | | age≥85 years | |
|  | Adjusted HR | *P* value | Adjusted HR | *P* value | Adjusted HR | *P* value | Adjusted HR | *P* value | Adjusted HR | *P* value | Adjusted HR | *P* value |
| Source of income | |  |  |  |  |  |  |  |  |  |  |  |
| Pension | Ref. | Ref. | Ref. | Ref. | Ref. | Ref. | Ref. | Ref. | Ref. | Ref. | Ref. | Ref. |
| Continuing to Work | 0.62(0.44, 0.88) | 0.007 | 0.74(0.42, 1.31) | 0.302 | 0.66(0.51, 0.84) | 0.001 | 0.61(0.44, 0.84) | 0.002 | 0.75(0.26, 2.15) | 0.598 | 1.18(0.60, 2.34) | 0.635 |
| Immediate Family | 1.24(0.92, 1.67) | 0.166 | 1.20(0.92, 1.57) | 0.169 | 1.20(0.97, 1.49) | 0.095 | 1.19(1.02, 1.40) | 0.031 | 1.23(0.66, 2.28) | 0.517 | 1.09(0.90, 1.33) | 0.369 |
| Others or Subsidy | 0.76(0.45, 1.29) | 0.314 | 1.51(1.09, 2.10) | 0.012 | 1.13(0.85, 1.50) | 0.390 | 1.35(1.12, 1.63) | 0.001 | 1.15(0.54, 2.44) | 0.710 | 1.15(0.93, 1.42) | 0.186 |
| Medical insurance | |  |  |  |  |  |  |  |  |  |  |  |
| None | Ref. | Ref. | Ref. | Ref. | Ref. | Ref. | Ref. | Ref. | Ref. | Ref. | Ref. | Ref. |
| UBMI | 1.07(0.79, 1.44) | 0.660 | 0.85(0.66, 1.09) | 0.199 | 0.84(0.68, 1.03) | 0.099 | 0.96(0.83, 1.11) | 0.545 | 1.01(0.54, 1.86) | 0.987 | 0.93(0.79, 1.09) | 0.351 |
| NRCMS | 1.04(0.81, 1.35) | 0.742 | 0.94(0.75, 1.18) | 0.600 | 0.87(0.75, 1.01) | 0.062 | 0.92(0.84, 1.02) | 0.097 | 0.90(0.66, 1.22) | 0.504 | 0.89(0.80, 0.98) | 0.021 |
| Commercial or Others | 0.72(0.39, 1.33) | 0.297 | 1.00(0.64, 1.55) | 0.996 | 0.73(0.49, 1.10) | 0.136 | 1.02(0.81, 1.29) | 0.842 | 0.55(0.19, 1.61) | 0.276 | 1.04(0.84, 1.28) | 0.735 |
| Community Support | |  |  |  |  |  |  |  |  |  |  |  |
| Don’t know or None | Ref. | Ref. | Ref. | Ref. | Ref. | Ref. | Ref. | Ref. | Ref. | Ref. | Ref. | Ref. |
| Daily Care | 1.22(0.76, 1.98) | 0.410 | 1.36(0.89, 2.08) | 0.157 | 0.90(0.63, 1.27) | 0.54 | 1.11(0.88, 1.38) | 0.375 | 0.90(0.47, 1.72) | 0.752 | 1.17(0.94, 1.46) | 0.156 |
| Socializing | 1.35(0.94, 1.95) | 0.109 | 1.09(0.76, 1.59) | 0.632 | 0.90(0.65, 1.25) | 0.532 | 1.09(0.90, 1.33) | 0.365 | 2.87(0.95, 8.63) | 0.061 | 1.07(0.86, 1.33) | 0.538 |
| Legal Needs | 1.04(0.80, 1.36) | 0.767 | 1.12(0.86, 1.46) | 0.411 | 1.02(0.88, 1.19) | 0.781 | 0.97(0.86, 1.09) | 0.619 | 1.35(0.94, 1.95) | 0.105 | 1.03(0.91, 1.18) | 0.612 |
| Daily Care & Socializing | 0.79(0.35, 1.79) | 0.575 | 1.26(0.73, 2.16) | 0.409 | 0.97(0.61, 1.55) | 0.908 | 1.14(0.86, 1.52) | 0.361 | 2.06(0.62, 6.85) | 0.240 | 1.12(0.85, 1.47) | 0.411 |
| All Above | 0.98(0.63, 1.52) | 0.915 | 0.80(0.44, 1.45) | 0.462 | 1.13(0.84, 1.52) | 0.425 | 0.90(0.70, 1.16) | 0.410 | 1.74(0.76, 4.02) | 0.192 | 1.05(0.82, 1.34) | 0.710 |
| Live Alone |  |  |  |  |  |  |  |  |  |  |  |  |
| No | Ref. | Ref. | Ref. | Ref. | Ref. | Ref. | Ref. | Ref. | Ref. | Ref. | Ref. | Ref. |
| Yes | 1.37(1.06, 1.79) | 0.017 | 0.93(0.74, 1.17) | 0.555 | 1.07(0.93, 1.24) | 0.348 | 0.87(0.78, 0.96) | 0.008 | 0.85(0.56, 1.28) | 0.429 | 0.91(0.80, 1.04) | 0.173 |

Abbreviations: UBMI: Urban Basic Medical Insurance; NRCMS: New Rural Cooperative Medical Scheme.
